# Multi-parametric MRI phenotype with trustworthy machine learning for differentiating CNS demyelinating diseases

**DOI:** 10.1186/s12967-021-03015-w

**Published:** 2021-09-06

**Authors:** Jing Huang, Bowen Xin, Xiuying Wang, Zhigang Qi, Huiqing Dong, Kuncheng Li, Yun Zhou, Jie Lu

**Affiliations:** 1 Department of Radiology and Nuclear Medicine, Xuanwu Hospital, Capital Medical University, No.45 Changchun Street, Xuanwu District, Beijing, 100053 China; 2 School of Computer Science, The University of Sydney, Building J12/1 Cleveland Street, Sydney, NSW 2006 Australia; 3 Beijing Key Laboratory of Magnetic Resonance Imaging and Brain Informatics, Capital Medical University, Beijing, China; 4 Department of Neurology, Xuanwu Hospital, Capital Medical University, Beijing, China; 5 Mallinckrodt Institute of Radiology, Washington University in St. Louis School of Medicine, St. Louis, MO 63110 USA

**Keywords:** Multiple sclerosis, Neuromyelitis optica, MRI, Radiomics, Machine learning

## Abstract

**Background:**

Misdiagnosis of multiple sclerosis (MS) and neuromyelitis optica (NMO) may delay the treatment, resulting in poor prognosis. However, the precise identification of these two diseases is still challenging in clinical practice. We aimed to evaluate the value of quantitative radiomic features extracted from the brain white matter lesions for differential diagnosis of MS and NMO.

**Methods:**

We recruited 116 CNS demyelinating patients including 78 MS, and 38 NMO. Three neuroradiologists performed visual differential diagnosis based on brain MRI for comparison purpose. A multi-level scheme was designed to harness the selection of discriminative and stable radiomics features extracted from brain while mater lesions in T1-MPRAGE, T2 sequences and clinical factors. Based on the imaging phenotype composed of the selected radiomic and clinical features, Multi-parametric Multivariate Random Forest (MM-RF) model was constructed and verified with both 10-fold cross-validation and independent testing. Result interpretation was provided to build trust in diagnostic decisions.

**Results:**

Eighty-six patients were randomly selected to form the training set while the rest 30 patients for independent testing. On the training set, our MM-RF model achieved accuracy 0.849 and AUC 0.826 in 10-fold cross-validation, which were significantly higher than clinical visual analysis (0.709 and 0.683, *p* < 0.05). In the independent testing, the MM-RF model achieved AUC 0.902, accuracy 0.871, sensitivity 0.873, specificity 0.869, respectively. Furthermore, age, sex and EDSS were found mildly correlated with the radiomic features (*p* of all < 0.05).

**Conclusions:**

Multi-parametric radiomic features have potential as practical quantitative imaging biomarkers for differentiating MS from NMO.

**Supplementary Information:**

The online version contains supplementary material available at 10.1186/s12967-021-03015-w.

## Background

Multiple sclerosis (MS) and Neuromyelitis optica (NMO) are demyelinating diseases of the central nervous system (CNS), which are the most common causes of neurological disability in young people [1]. In the pathogenesis of these two diseases, a variety of immune-related molecules and pathways are different [2]. In clinical practice, the differential diagnosis of these two diseases is still challenging. It is reported that around 30% of the misdiagnosed MS cases were diagnosed as NMO [3]. There are several factors contributing to the difficulty of differential diagnosis, e.g., they share overlapped features in clinical symptoms such as myelitis, optic neuritis [1, 4], and laboratory examinations (30% of the NMO patients had the same negative results of NMO immunoglobulin G as MS patients [5]). Misdiagnosis can lead to unprecise treatment and sometimes even exacerbation of the disease, as the treatment for MS differs greatly from that of NMO [6].

Magnetic resonance imaging (MRI) is routinely used in the differential diagnosis of MS and NMO; however, its specificity is limited because partial lesions in brain white matter of two diseases share similar lesion appearance, location distribution, and signal characteristics on MRI [3, 7–9]. In addition to similar neuroimaging characteristics, another common cause of MS misdiagnosis is the subjective visual observation and analysis, such as misinterpretation and misapplication of abnormal MRI findings as suggested by Solomon et al. [3]. Therefore, it is in high demand for a quantitative, repeatable and objective measurement for the differential diagnosis.

Radiomics is an emerging field with a surge of interest due to its capabilities to extract quantitative biomedical imaging “markers” for automated objective diagnosis [10, 11], and potentially to foster individualized diagnosis. Empowered with machine learning and deep learning, radiomics methodology mines the valuable information underlying imaging that could be beyond the perception capacity of human beings and has been successfully applied for differential diagnosis of other CNS diseases [12–14]. Though radiomic models are able to produce promising diagnostic results with higher accuracy, clinicians often find it difficult to interpret the results from machine learning models. To be clinically applicable, there is an urgent need to address the “lacking interpretability” problem [15].

In this study, we aim to investigate a quantitative and objective MRI-based radiomics platform, equipped with individualized result interpretation, to provide clinicians with trustworthy assistance for diagnostic differentiation.

## Methods

### Subjects and MRI acquisition

This study was approved by the institutional review board of Xuanwu Hospital, Capital Medical University, and written informed consent was obtained from all participants. Totally 116 participants were recruited including 78 relapsing–remitting MS and 38 NMO patients. The impact of unbalanced data was comprehensively assessed with metrics including AUC, diagnosis accuracy, sensitivity and specificity. The patients were composed of two cohorts with different MRI protocols as shown in Additional file 1: Table S1. The first cohort included patients from April 2004 to December 2004 who underwent brain scanning on 1.5 T MRI (Sonata; Siemens Medical Systems, Erlangen, Germany) with an 8-channel head coil. The second cohort included patients from November 2009 to April 2014 who had brain scanning on 3 T MRI (Siemens Magnetom Trio Tim System, Munich, Germany). As a proportion of patient cohorts were recruited before the introduction of the new MS and NMO criteria, our diagnosis of MS and NMO was based on the 2010 McDonald criteria, and the revised NMO diagnostic criteria, respectively [16, 17]. None of these patients had been treated with medications within three months before the MRI was obtained. The demographic and clinical characteristics including Expanded Disability Status Scale (EDSS) score [18] and Disease Duration of the patients were recorded.

### Clinical MRI review

Three neuroradiologists with 5, 7, and 10 years of MRI reading experience were involved in the visual assessment of the brain lesion and differential classification of MS and NMO patients. The assessment was based on T1-MPRAGE and T2 MRI sequences, while the clinical data (age, sex, disease duration and EDSS score) was allowed to refer during the assessment. Each neuroradiologist produced a diagnostic result for each patient based on their own clinical experience. In case of any discrepancy, it shall be jointly reviewed to reach an agreement. The assessments such as AUC, diagnosis accuracy, sensitivity and specificity were calculated.

### Radiomic analysis overview

**Fig. 1:**
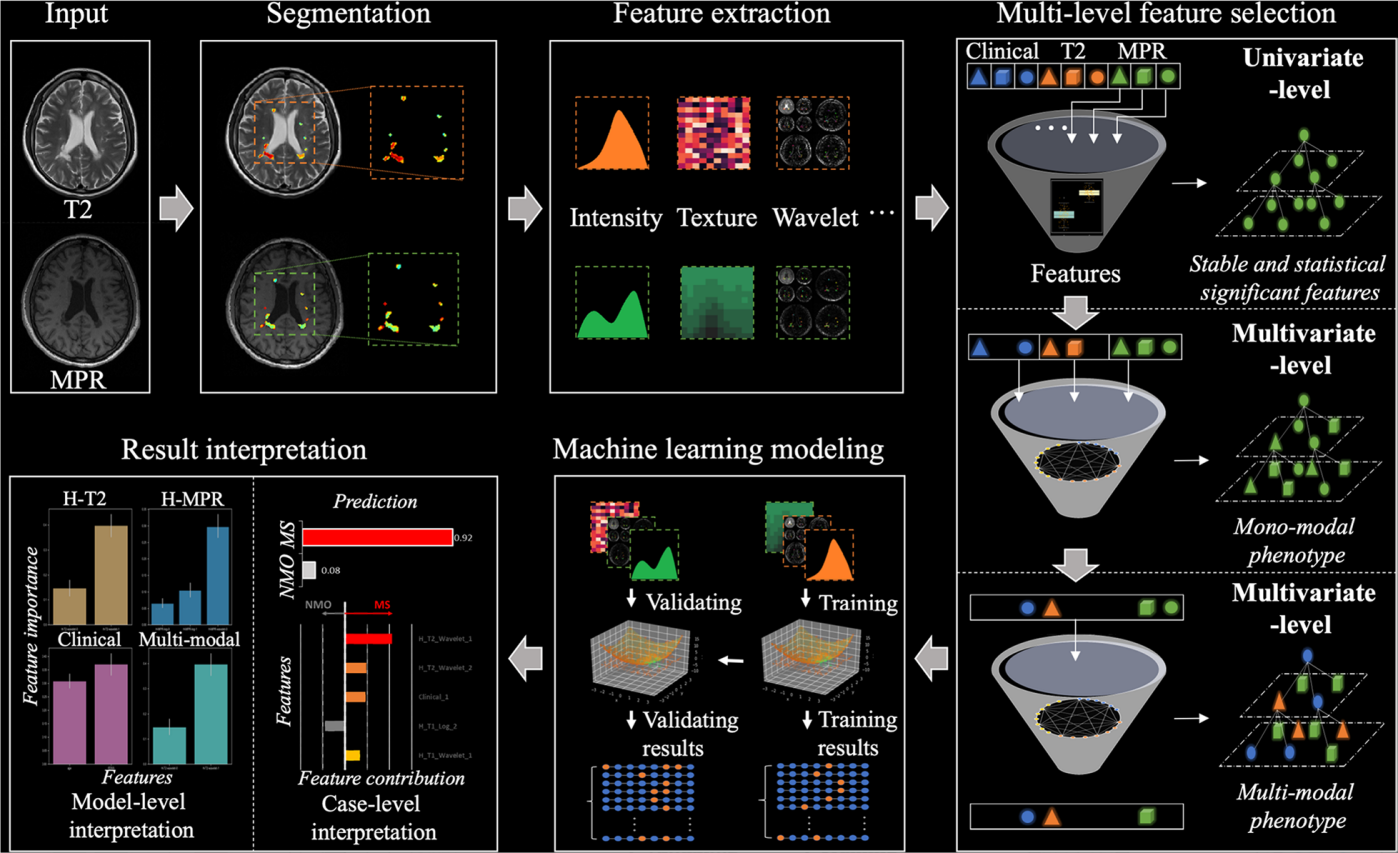
Flowchart of our radiomics pipeline. *MPR* magnetization-prepared rapid gradient-echo

Radiomic analysis framework was composed of five main modules, including image segmentation, feature extraction, feature selection (phenotype building), machine learning modeling, and quantitative interpretation of results. An overview was provided in Fig. 1.

### Image processing and feature extraction

Marking of hyperintense brain lesions volume on T2 sequences was performed by a neuroradiologist with more than 9 years of experience (J.H.) using MRIcro software (https://people.cas.sc.edu/rorden/mricro/), and validated by a senior neuroradiologist (Z.Q.), who had more than 20 years of experience. The volume of interests (VOIs) delineated on T2 sequence were mapped to T1-MPRAGE sequence through rigid image registration to automatically obtain the corresponding VOIs from T1-MPRAGE sequence.

Then, from the VOIs of both MRI sequences, we extracted 1118 quantitative radiomic features for each sequence that embraced 18 intensity, 68 texture, 344 Laplace of Gaussian (LoG) features, and 688 wavelet features [19]. LoG and wavelet filters were applied before the texture feature extraction to reduce the impact of noise. After feature extraction, all features were standardized to be comparable in scale. Other descriptions and implementation details of radiomic features are described in Additional file 1: Appendix S1 and Table S2 respectively.

### Statistical analysis

#### Multi-level Feature Selection for imaging phenotype construction

Our Multi-level Feature Selection algorithm aimed to solve two key challenges in feature selection in the clinical context, including: (1) selecting relevant and discriminative features from high-dimensional small-sample multimodal data, and (2) mining robust features across MRI images with different imaging quality (e.g., MRI images with different magnetic field strength), which is often neglected by feature selection algorithms [20]. To address these two challenges, we design a Multi-level Feature Selection algorithm, composed of univariate-level and multivariate-level module, to jointly explore feature relevancy, robustness and discriminability.

In univariate-level module, we design a statistical filter scheme on the basis of Wilcoxon Rank-sum test to simultaneously select: (1) robust features by testing the statistical consistency across MRI with different image quality, and (2) relevant features through calculating statistical relevancy towards the outcome. Specifically, robust features across different magnetic field strength of MRI scanners (1.5 T and 3 T) were first selected with Wilcoxon test [21]. Then, discriminative features were selected by assessing whether there was a significant distribution difference between MS patients and NMO patients via Wilcoxon test [22].

In multivariate-level module, we propose a pyramid searching structure to first exploit intra-modal feature relationships and then explore inter-modality relationships. This pyramid searching scheme boosted feature discriminability and mining efficiency. In contrast, conventional feature selection often uses a flattened search space by concatenating all features for feature selection. In specific, Random Forest-based sequential forward selection (RF-SFS) was firstly employed to select discriminative features and construct preliminary phenotypes from T2, T1-MPRAGE and clinical features separately [23]. Then, a multi-parametric phenotype was constructed by further applying RF-SFS to a fused feature set of three preliminary phenotypes. The mathematical details of Multi-level Feature Selection were summarized in Additional file 1: Appendix S2.

### Classification analysis

To compare the diagnostic performance of preliminary phenotypes of T2, T1-MPRAGE and clinical and the multi-parametric phenotype, three preliminary Multivariate Random Forest models and Multi-parametric Multivariate Random Forest model (MM-RF) were constructed based on the corresponding phenotype respectively. To handle data imbalance, balanced bootstrap mechanism and balanced weight [24] were incorporated into the Random Forest model. Common evaluation metrics for imbalanced datasets were used for assessing the diagnostic performance of models, including the area under the receiver operating characteristic curve (AUC), accuracy, sensitivity, and specificity. The stability of diagnostic performance was assessed with the mean of relative standard deviation (RSD) of AUC. The lower the AUC_RSD value, the higher the stability.

1 $$ AUC\_RSD = \frac{AUC\_STD}{{AUC\_MEAN}} $$

To validate the feature selection, our Multi-level Feature Selection algorithm was compared with eight state-of-the-art feature selection algorithms that were commonly used in radiomics studies [25–27]. These algorithms included three Filter Selection methods (Wilcoxon filter [28], Anova filter [29], mRMR filter [30]), two Wrapper Selection methods (RF wrapper [31], and SFS wrapper [32]), and three Embedded Selection methods (Lasso [33], ElasticNet [34], RFE_SVM [35]).

### Validation

To rigorously validate the diagnostic performance of the imaging phenotypes, both 10-fold cross-validation on the training set and independent validation on the testing set were computed. From a total of 116 patients, 86 patients were randomly selected to form the training set, while the rest 30 patients was used for independent testing. Bootstrapping with 1000 times resampling was used in the independent validation.

All statistical analysis was two-sided, with the significance level of 0.05. Multi-level statistical analysis was performed with “scipy”, “sklearn”, “mlxtend”, “mifs”, “imblearn” modules in Python 3.6. Correlation analysis was performed in R 3.5.1.

### Quantitative interpretation of results

Lack of interpretability is a key challenge as the basis for trustworthy decision making [36]. To provide quantitative interpretation, we utilized SHAP method [37] to analyse the differential decision from our MM-RF model at both individual-level and model-level. The individual-level interpretation explained the output of an individual prediction by visualizing the important features in the phenotype and unveiling their importance for discrimination decisions. The model-level interpretation computed the average feature importance across all patients and revealed the relationship between the feature value and its importance.

## Results

### Demographics and clinical and MRI characteristics

Seventy-eight relapsing–remitting MS patients (mean age ± SD: 36.5 years ± 10.0), 38 NMO patients (mean age ± SD: 40.9 years ± 11.7) participated in this study. The percentages of males out of all patients were 34.6%, 18.4%, respectively. There were no significant differences in sex and age between MS and NMO patients. NMO group showed a trend towards higher EDSS score than the MS group (*p* = 0.005). Other demographic characteristics of the participants were provided in Table 1.

**Table 1:** Patient characteristics

Characteristics	3 T MRI cohort		1.5 T MRI cohort		Both cohorts		*P*
	MS (n = 38)	NMO (n = 30)	MS (n = 40)	NMO (n = 8)	MS (n = 78)	NMO (n = 38)	
Age, year, mean ± SD	35.7 ± 9.5	41.5 ± 10.8	37.4 ± 10.6	38.5 ± 15.4	36.5 ± 10.0	40.9 ± 11.7	0.053^a^
Female/male	25/13	23/7	26/14	8/0	51/27	31/07	0.114^b^
EDSS, mean ± SD	3.1 ± 1.7	3.8 ± 1.7	2.8 ± 1.4	4.1 ± 1.6	2.9 ± 1.5	3.8 ± 1.6	0.005^a^
Disease duration, month, mean ± SD	62.5 ± 56.4	61.7 ± 56.3	50.8 ± 50.9	84.0 ± 67.0	56.8 ± 54.1	66.4 ± 58.4	0.396^a^

*MS* multiple sclerosis, *NMO* neuromyelitis optica, *EDSS* expanded disease severity scale, *SD* standard deviation

^a^Two-sample *t*-test

^b^Chi-squared test

### Clinical visual analysis

The AUC of the visual analysis was 0.683 with 95% Confidence Interval (CI) 0.571–0.789. The visual analysis successfully diagnosed 61 out of 86 patients, with accuracy reaching 0.709 (95% CI 0.616–0.802). In the misdiagnosed 25 cases, 10 NMO patients were misdiagnosed as MS while 15 MS patients were misdiagnosed as NMO. Its sensitivity and specificity were 0.615 and 0.750 respectively.

### Radiomic feature selection and phenotype construction

**Fig. 2:**
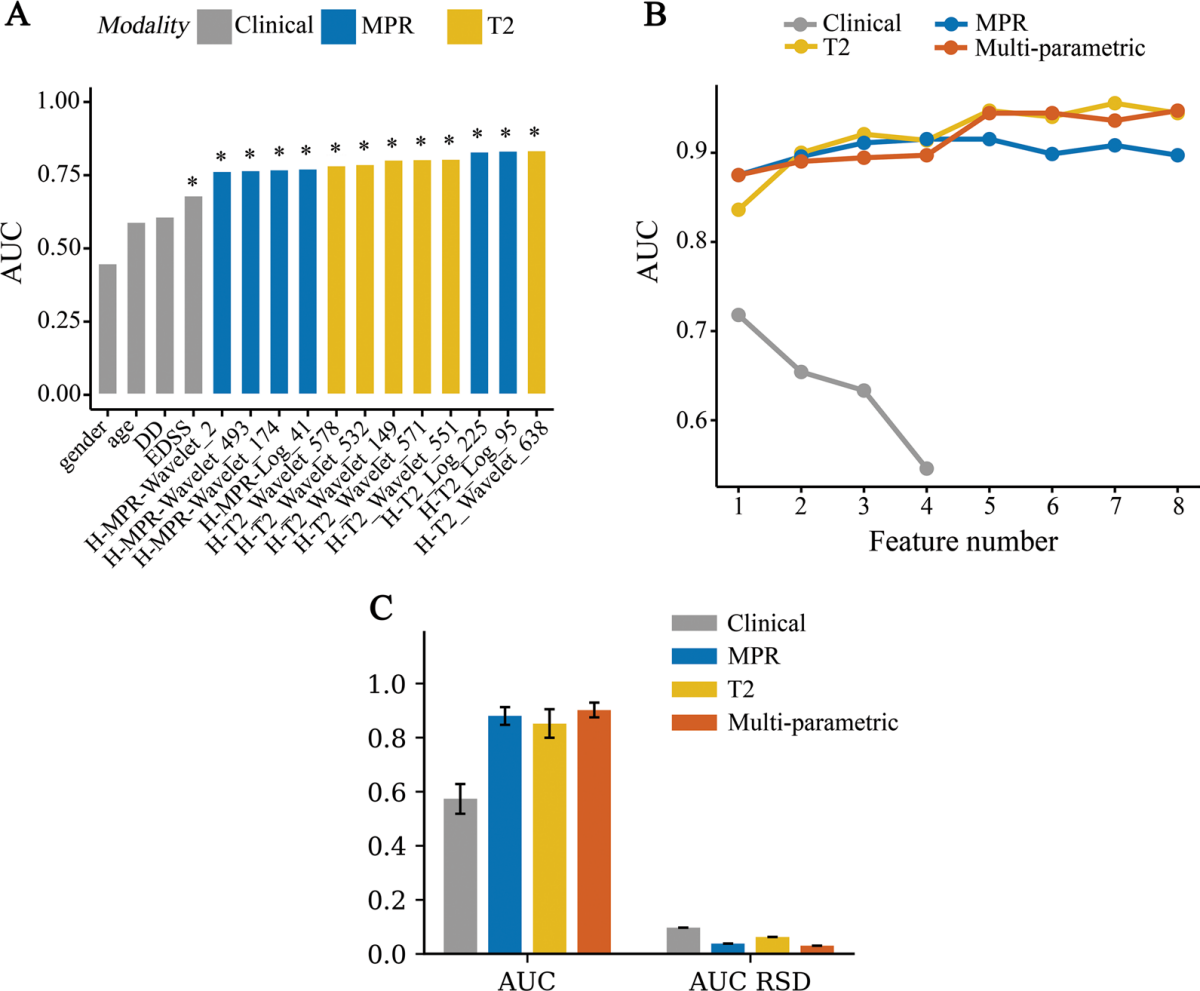
Results of Multi-level Feature Selection and phenotype testing. *AUC* area under the curve, *RSD* relative standard deviation, *MPR* magnetization-prepared rapid gradient-echo. **A** Results of univariate feature selection and analysis. Top six T2 and T1-MPRAGE features and four clinical factors were selected and ranked according to AUC. The asterisk (*) represents statistical significance (p < 0.05) with Wilcoxon test. **B** Results of multivariate feature selection. The algorithm selected a subset of features having the highest AUC. The arrows indicate the stoping points where the highest AUC was achieved. **C** Comparison of diagnostic performance of T2, T1-MPRAGE, clinical phenotypes and the multi-parametric phenotype in the independent testing

Figure 2A demonstrates the differentiation ability of top univariately selected features and clinical factors. It shows that T2 and MPR radiomics features generally achieved higher AUC than clinical features at univariate level. Figure 2B illustrates the process of multivariate SFS feature selection, which shows multivariate imaging phenotypes had higher discriminability compared with clinical features. The multi-parametric phenotype was established with three T2, four T1-MPRAGE and one clinical feature. These eight features and their corresponding feature identification number (id) were H-T2-waveletHHL-glcm-Idn (116), H-T2-log2-glcm-Autocorrelation (18), H-T2-waveletLLH-glcm-JE (551), H-MPR-waveletLHL-glszm-GLNU (500), H-MPR-log4-gldm-SDLGLE (225), H-MPR-log3-firstorder-Median (95), H-MPR-log5-glcm-Idmn (287), and EDSS. Other details of feature selection results are summarized in Additional file 1: Appendix S3.

### Evaluation of multi-parametric phenotype in terms of discriminative ability

The multi-parametric phenotype was evaluated with both 10-fold cross validation and independent testing. In cross-validation, the multi-parametric phenotype achieved AUC 0.826 (95% CI 0.732–0.912), which was significantly higher than that of visual analysis (*p* = 0.016). The diagnostic accuracy was 0.849 (95% CI 0.767–0.919), higher than that of visual analysis (*p* = 0.008). Its sensitivity and specificity were 0.769 and 0.883, respectively.

In the independent testing, the multi-parametric phenotype based on Random Forest model achieved AUC 0.902 ± 0.027, which was higher than the performance of preliminary T2, T1-MPRAGE and clinical phenotypes (Fig. 2C). The diagnostic AUC of T2, T1-MPRAGE and clinical phenotype was 0.852 ± 0.053, 0.880 ± 0.033 and 0.573 ± 0.055, respectively. Figure 2C also illustrates that multi-parametric phenotype achieved better stability of diagnostic performance. Other assessments of the multi-parametric phenotype such as diagnostic accuracy, sensitivity and specificity were 0.871 ± 0.044, 0.873 ± 0.083 and 0.869 ± 0.051, respectively, as reported in Table 2. To assess the impact of 3 T and 1.5 T MRI, a further experiment showed that high diagnostic accuracy was achieved by both 3 T (0.856 ± 0.046) and 1.5 T (0.976 ± 0.074) cohort (p < 0.05).

**Table 2:** Diagnostic performance of our method compared with 8 SOTA feature selection algorithms

Category	Method	Roc_auc	Sensitivity	Specificity	Accuracy
Filter	Wilcoxon [28]	0.625 ± 0.120	0.604 ± 0.145	0.585 ± 0.137	0.593 ± 0.104
Filter	Anova [29]	0.879 ± 0.037	0.883 ± 0.042	0.726 ± 0.067	0.789 ± 0.036
Filter	mRMR [30]	0.846 ± 0.041	0.823 ± 0.091	0.754 ± 0.076	0.782 ± 0.050
Wrapper	RF [31]	0.846 ± 0.041	0.828 ± 0.091	0.750 ± 0.075	0.781 ± 0.048
Wrapper	SFS [32]	0.858 ± 0.038	0.782 ± 0.073	0.829 ± 0.121	0.810 ± 0.067
Embedded	Lasso [33]	0.873 ± 0.050	0.884 ± 0.046	0.737 ± 0.087	0.796 ± 0.056
Embedded	ElasticNet [34]	0.850 ± 0.064	0.868 ± 0.070	0.719 ± 0.135	0.779 ± 0.086
Embedded	RFE [35]	0.814 ± 0.072	0.846 ± 0.058	0.612 ± 0.141	0.705 ± 0.090
Ours	Clinical	0.573 ± 0.055	0.716 ± 0.190	0.446 ± 0.179	0.554 ± 0.070
Ours	T2 MRI	0.852 ± 0.053	**0.887 ± 0.067**	0.733 ± 0.057	0.795 ± 0.045
Ours	T1-MPR MRI	0.880 ± 0.033	0.798 ± 0.102	0.859 ± 0.073	0.835 ± 0.060
Ours	Multi-parametric MRI	**0.902 ± 0.027**	0.873 ± 0.083	**0.869 ± 0.051**	**0.871 ± 0.044**

The Bolded value indicates the highest value in each column

*AUC* area under the curve, *Anova* analysis of variance, *mRMR* maximum relevance minimum redundancy, *RF* random forest, *SFS* sequential forward selection, *Lasso* least absolute shrinkage and selection operator, *RFE* recursive feature elimination

### Evaluation of Multi-level Feature Selection compared with eight state-of-the-art methods

Table 2 shows the performance comparison of our Multi-level Feature Selection methods with eight state-of-the-art methods. From a combined feature pool of T2, T1-MPR and clinical features, these comparison methods selected at most eight features as in our method, and were evaluated with the same Random Forest model as ours. The experimental results in Table 2 showed that our Multi-level Feature Selection algorithm outperformed these SOTA methods in comparison. Our multiparametric phenotype achieved highest AUC 0.902 ± 0.027, followed by our MPR phenotype (AUC 0.880 ± 0.033) and Anova Filter (AUC 0.879 ± 0.037).

### Case studies for individual-level interpretation

**Fig. 3:**
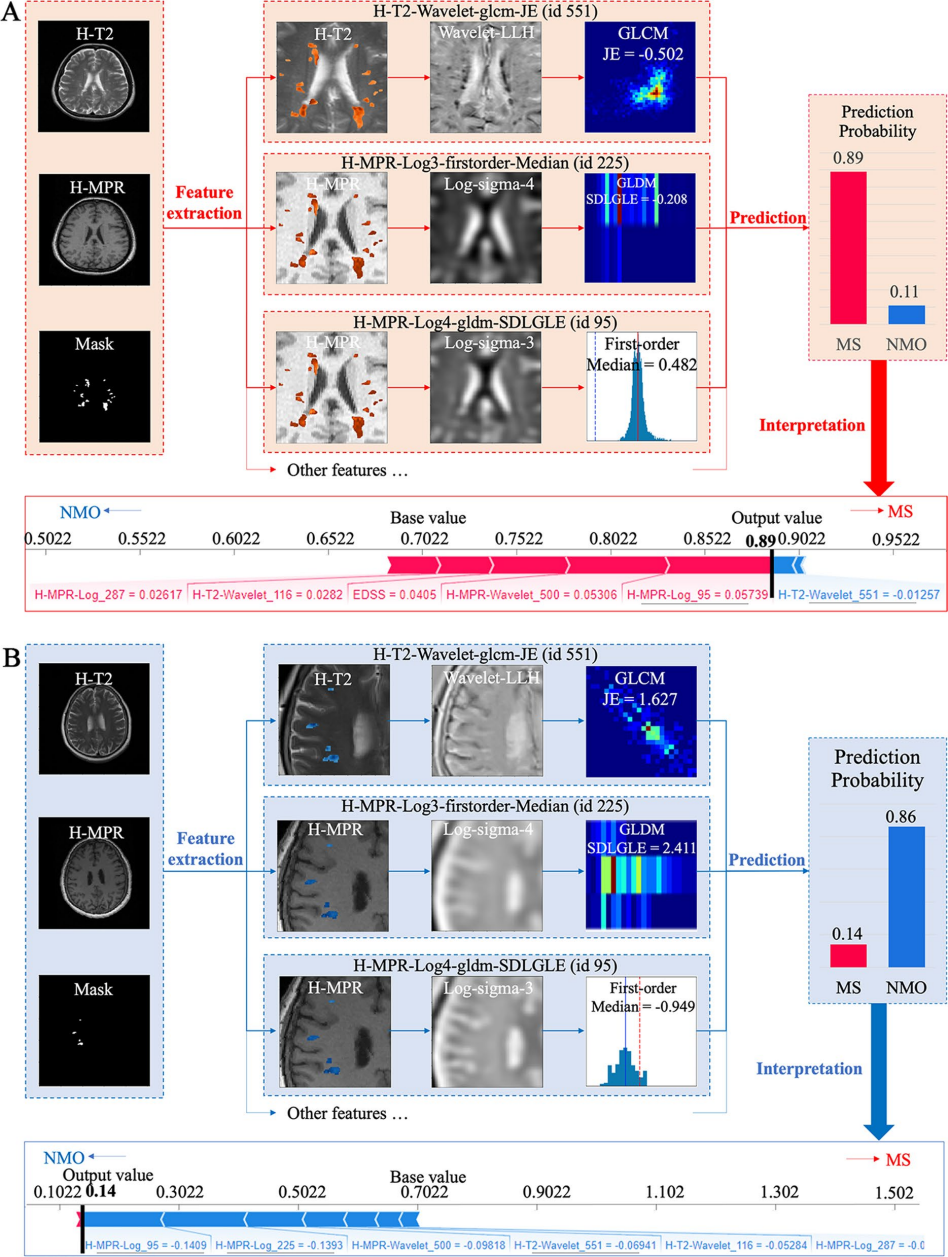
Results of individual-level interpretation. *LoG* Laplace of Gaussian, *MPR* magnetization-prepared rapid gradient-echo; The interpretation of two representative cases from MS (case **A**) and NMO (case **B**) was illustrated. For each case, visualization of three key radiomic features was provided. The classification results were computed with Random Forest. Lastly, the classification results were explained by revealing feature contribution

As illustrated in Fig. 3, the two selected cases included: (a) an MS case, and (b) an NMO case, whose lesions were difficult to differentiate due to similar lesion location and signal characteristics. With extracted phenotype from VOIs in the MR images, our MM-RF classified the cases correctly with 89% confidence for MS case and NMO case with 86% confidence, respectively. For the MS case, our case-level interpretation revealed that H-MPR-log3-firstorder-Median, H-MPR-waveletLHL-glszm-GLNU, and EDSS were the three most significant contributors for accurate classification, with 29.86%, 27.61% and 21.07% contribution, respectively. As a contrast, for the NMO case, three T1-MPRAGE features (H-MPR-log3-firstorder-Median, H-MPR-log4-gldm-SDLGLE, H-MPR-waveletLHL-glszm-GLNU) were three major contributors, contributing 25.06%, 24.78%, 17.46% towards the correct decision.

### Model-level result interpretation

**Fig. 4:**
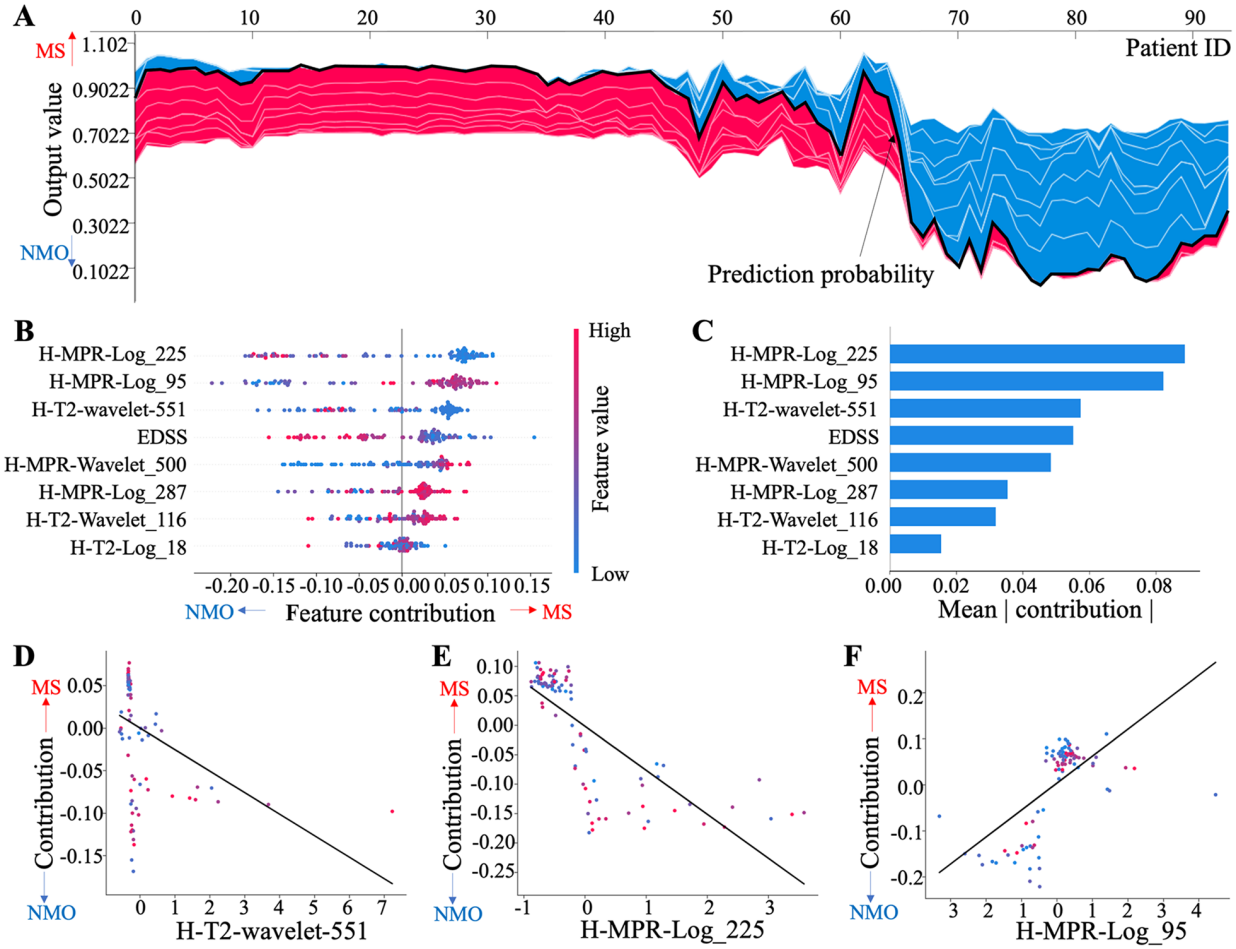
Results of model-level interpretation. *LoG* Laplace of Gaussian, MPR magnetization-prepared rapid gradient-echo, *EDSS* expanded disease severity scale; **A** Visualization of feature contribution for all individual cases. Each vertical line corresponds to interpretation for individual diagnosis. Red represents a diagnosis of MS, blue for NMO. **B** Summary plot of feature contribution for all individual cases. **C** Mean contribution of features in the multi-parametric phenotype. **D**–**F** Relationship between feature value and feature importance. The straight lines were obtained through curve fitting with linear regression

Model-level interpretation investigated the feature importance and the relationship between feature value and its importance from the perspective of all patients. Figure 4A shows case-level interpretation results of all patients in one graph, which visualizes how feature contributions differ for different cases. Of all eight features, H-MPR-log4-gldm-SDLGLE, H-MPR-log3-firstorder-Median, and H-T2-waveletLLH-glcm-JE were the top three important features in the model decision making, as shown in Fig. 4B, C. In terms of relationship between the feature value and its importance, there was a negative linear relationship for H-T2-waveletLLH-glcm-JE (Fig. 4D) and H-MPR-log4-gldm-SDLGLE (Fig. 4E), and a positive linear relationship for H-MPR-log3-firstorder-Median (Fig. 4F).

### Association of selected radiomic features with clinical variables

**Fig. 5:**
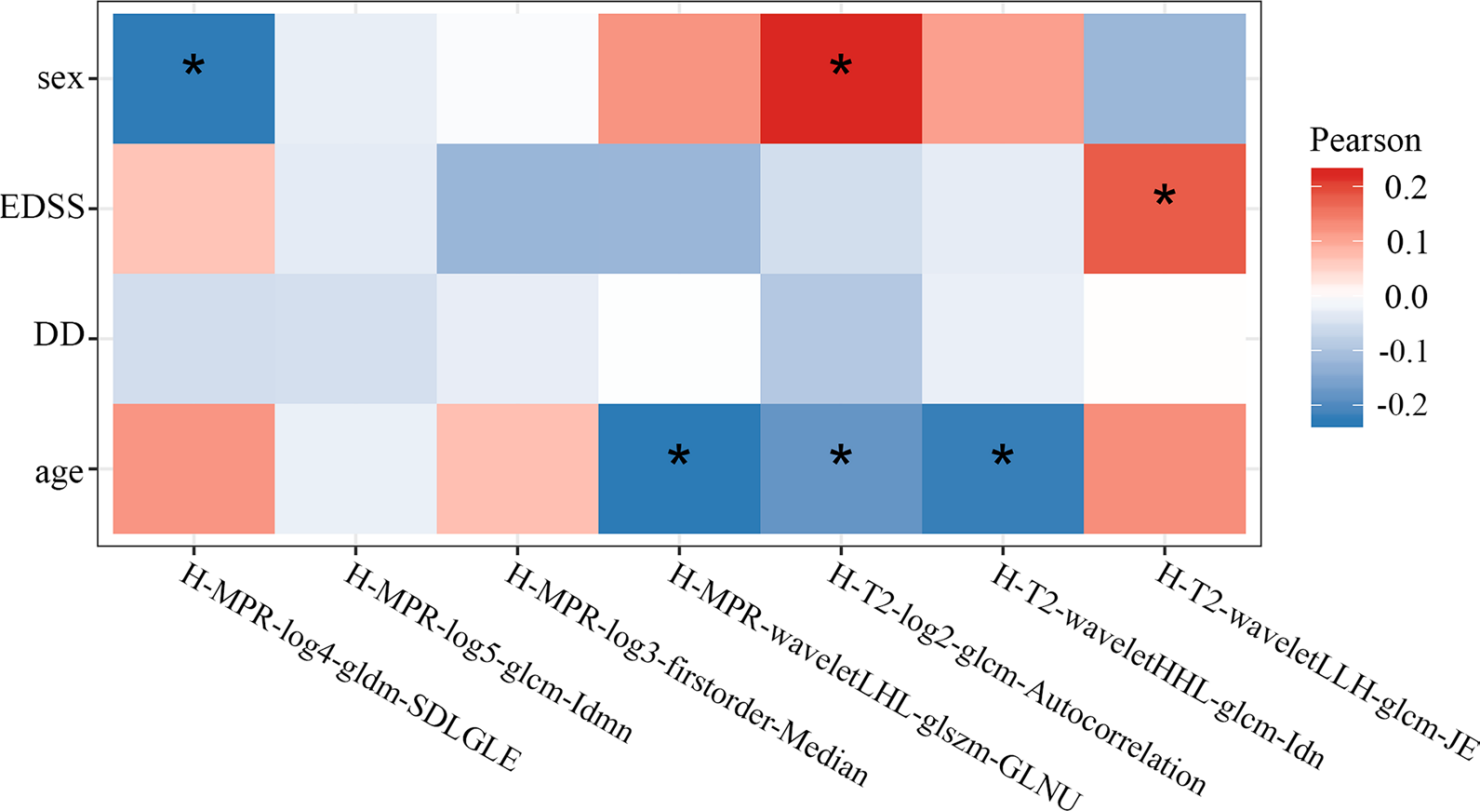
Results of correlation analysis. *EDSS* expanded disease severity scale, DD disease duration, *LoG* Laplace of Gaussian. Correlation matrix was computed for seven radiomic features in the multi-parametric phenotype and four clinical features (age, sex, DD, EDSS). Red and blue color show the positive and negtive correlation respectively. The asterisk (*) represents statistical significance

As showed in Fig. 5, sex was found significantly negatively correlated with H-MPR-log4-gldm-SDLGLE (*p* = 0.008), while positively correlated with H-T2-log2-glcm-Autocorrelation (*p* = 0.035). EDSS scores was significantly positively correlated with H-T2-waveletLLH-glcm-JE (*p* = 0.036). Age was significantly negatively correlated with H-MPR-waveletLHL-glszm-GLNU (*p* = 0.007), and H-T2-waveletHHL-glcm-Idn (*p* = 0.010), and H-T2-log2-glcm-Autocoorelation (*p* = 0.035).

## Discussion

In this research, we extracted the imaging phenotype from multi-parametric MRI sequences with the machine learning framework for automated differentiating MS from NMO, which provided an additional reference for timely differential diagnostic decision making. The major findings of this study include: (1) our multi-parametric phenotype was able to achieve high differential diagnostic performance, generalizability and robustness, mined by our designed Multi-level Feature Selection algorithm; (2) our radiomics platform provided individualized differential diagnosis and interpretation which was illustrated with a case study; and (3) the correlation between radiomic and clinical features was revealed to enhance trust in radiomic features.

The first finding of our study is that the multi-parametric phenotype demonstrated high differential diagnostic performance, which statistically outperformed visual analysis in terms of AUC (0.826 vs. 0.683, *p* = 0.016), and the diagnosis accuracy (0.849 vs. 0.709, *p* = 0.008) in 10-fold cross-validation. The accuracy of clinical visual analysis in our study complied with the studies [38–42] with the reported accuracy ranging from 0.573 to 0.739. In this study, doctors misdiagnosed about 25% of patients with MS as NMO, similar to the previous study [2], which justified the machine learning model provide valuable assistance for clinical decisions. Remarkably, the multi-parametric phenotype demonstrated the highest discriminative ability (AUC 0.902 ± 0.027) in the independent testing, outperforming the discriminative performance of the T2 (AUC 0.852), T1-MPRAGE (AUC 0.880) and clinical phenotypes (AUC 0.573). It indicates that the multi-parametric phenotype successfully fused pathological characteristics such as the information about edema, demyelination in T2 images, axonal damage in T1-MPRAGE images [43] and clinical information. This finding is consistent with previous studies in the differential diagnosis of brain tumors where the model embracing MRI-based radiomic features and clinical features can achieve the highest classification accuracy [44]. The present study, for the first time, constructs a multi-parametric phenotype including T2, T1-MPRAGE and clinical information for differentiating MS from NMO.

Our Multi-level Feature Selection algorithm outperformed SOTA feature selection methods because the proposed algorithm comprehensively considered: (1) feature robustness across MRI images with different magnetic fields strength, (2) feature relevancy towards the outcome, and (3) intra-modal and inter-modal feature discriminability. Comparatively, filter methods [28–30] select features using the defined feature relevancy such as mutual information; however, these methods might not take account of the interaction with the learning algorithm, and hence feature discriminability might not be optimized [45]. To address this issue, both Wrapper [31, 32] and Embedded [33–35] methods involve learning algorithms to assess the predictive performance of feature combinations. However, these methods might be prone to overfitting due to the dependency of the learning algorithm [46]. In contrast, our univariate-level selection selected robust and relevant features based on Wilcoxon testing, which addressed feature generalizability issue across different MRI imaging qualities (as analysed in [20]) and facilitated alleviating the risk of overfitting. Further, our multivariate-level selection boosted feature discriminability by exploiting intra-modal feature interaction and inter-modality interaction using a pyramid search structure. Due to the reduced risk of overfitting and boosted feature discriminability, our algorithm outperformed SOTA methods.

Secondly, individual-level interpretation was provided to articulate machine learning-based decision making for individual patients and thus to facilitate the trustworthy and individualized differential diagnosis. It was achieved by graphical visualization of important features and unveiling of quantitative contribution of features in the machine learning models which facilitates understanding on both radiomic features and model decisions, as illustrated in case studies in Fig. 3A, B. Specifically, for the case in Fig. 3A, this patient was correctly classified as MS with 89% confidence by our phenotype, in which the top three important features were two T1-MPRAGE features and EDSS. And for the case in Fig. 3B, three T1-MPRAGE radiomic features were major contributors. Interpretability enables doctors to gain insight why the diagnosis is made, thus assisting clinicians to provide precise differential diagnosis [47, 48].

Furthermore, the trust in the radiomic features was enhanced by revealing its connection with the clinical information. Mild correlations were observed between the radiomic features and clinical features (age, sex, and EDSS). Interestingly, we found that one T2 feature was related to EDSS (Fig. 5). The reason underlying the correlation between EDSS and T2 features might be that EDSS was found correlated with lesion load and brain atrophy [49, 50], while T2 and T1-MPRAGE images could also reflect the information of lesion and brain structure, respectively. As a result, we may potentially use radiomic features to objectively and conveniently assist EDSS in evaluating the treatment and disability management in the future. Although the above assumptions are preliminary, our study provides a perspective for understanding the clinical significance of radiomic features, in response to the urgent clinical need [51].

We suggest that our multi-parametric phenotype may serve as an objective, quantitative tool to assist clinical differential diagnosis of MS and NMO. Compared with the current diagnostic criteria of these two diseases using MR, our phenotype holds advantages in three aspects: (1) Instead of diagnosis by naked eye based on vague clinical experience, our multi-parametric phenotype provide a quantitative solution by feature extraction of medical images, thus help complement and clarify the current diagnostic criteria; (2) The phenotype reduces inter-observer variety and subjectivity because the whole system is highly automated; and (3) The current model can achieve high diagnostic accuracy with conventional MR sequences, which is simple and operable in clinical practice [52].

## Conclusion

Radiomic features extracted from T1-MPRAGE and T2 sequences are potential practical imaging biomarkers for differentiating the lesions of demyelinating diseases. They have been shown to be discriminative and robust in classifying the brain white matter lesions between MS and NMO. Effective interpretation of radiomic features coupled with machine learning methods can be used as an adjunct to traditional radiology to support the diagnostic process in clinical practice.

## Supplementary Information

**Additional file 1.** Appendix S1 describes radiomics features. Appendix S2 summarizes mathematical details of Multi-level Feature Selection. Appendix S3 shows results of feature selection. Table S1 describes imaging protocol for each cohort with 1.5T and 3T MRI. Table S2 shows the parameter settings for feature extraction.

## Data Availability

The datasets analyzed during the current study are available from the corresponding author on reasonable request.

## Abbreviations

AUC: Area under the curve
CNS: Central nervous system
DD: Disease duration
EDSS: Expanded disease severity scale
LoG: Laplace of Gaussian
MM-RF: Multi-parametric multivariate random forest
T1-MPRAGE: T1-magnetization-prepared rapid gradient-echo
MRI: Magnetic resonance imaging
MS: Multiple sclerosis
NMO: Neuromyelitis optica
RSD: Relative standard deviation
VOI: Volume of interest

## References

[1] Compston A, Coles A (2002). Multiple sclerosis. Lancet.

[2] Li Z, Liu YC, Jia AL, Cui YR, Feng J (2021). Cerebrospinal fluid cells immune landscape in multiple sclerosis. J Transl Med.

[3] Solomon AJ, Naismith RT, Cross AH (2019). Misdiagnosis of multiple sclerosis: impact of the 2017 McDonald criteria on clinical practice. Neurology.

[4] Solomon AJ, Bourdette DN, Cross AH, Applebee A, Skidd PM, Howard DB (2016). The contemporary spectrum of multiple sclerosis misdiagnosis: a multicenter study. Neurology.

[5] Wingerchuk DM, Lennon VA, Lucchinetti CF, Pittock SJ (2007). The spectrum of neuromyelitis optica. Lancet Neurol.

[6] Franca W, Lorenz G, Arsany H, Nicole K, Michael PH, Julia M (2018). Rebound after fingolim, od and a single daclizumab injection in a patient retrospectively diagnosed with NMO spectrum disorder-MRI apparent diffusion coefficient maps in differential diagnosis of demyelinating CNS disorders. Front Neurol.

[7] Carmosino MJ, Brousseau KM, Arciniegas DB, Corboy JR (2005). Initial evaluations for multiple sclerosis in a university multiple sclerosis center: outcomes and role of magnetic resonance imaging in referral. Arch Neurol.

[8] Liu S, Kullnat J, Bourdette D, Simon J, Kraemer DF, Murchison C (2013). Prevalence of brain magnetic resonance imaging meeting Barkhof and McDonald criteria for dissemination in space among headache patients. Mult Scler.

[9] McDonald WI, Compston A, Edan G, Goodkin D, Hartung HP, Lublin FD (2001). Recommended diagnostic criteria for multiple sclerosis: guidelines from the international panel on the diagnosis of multiple sclerosis. Ann Neurol.

[10] Li H, Xu C, Xin B, Zheng C, Zhao Y, Hao K (2019). (18)F-FDG PET/CT Radiomic analysis with machine learning for identifying bone marrow involvement in the patients with suspected relapsed acute leukemia. Theranostics.

[11] Gillies RJ, Kinahan PE, Hricak H (2016). Radiomics: images are more than pictures they are data. Radiology.

[12] Castillo D, Lakshminarayanan V, Rodríguez-Álvarez MJ (2021). MR images, brain lesions, and deep learning. Appl Sci.

[13] Kremer S, Renard F, Achard S, Lana-Peixoto MA, Palace J, Asgari N (2015). Use of advanced magnetic resonance imaging techniques in neuromyelitis optica spectrum disorder. JAMA Neurol.

[14] Qian Z, Li Y, Wang Y, Li L, Li R, Wang K (2019). Differentiation of glioblastoma from solitary brain metastases using radiomic machine-learning classifiers. Cancer Lett.

[15] Liu Z, Wang S, Dong D, Wei J, Fang C, Zhou X (2019). The Applications of radiomics in precision diagnosis and treatment of oncology: opportunities and challenges. Theranostics.

[16] Polman CH, Reingold SC, Banwell B, Clanet M, Cohen JA, Filippi M (2011). Diagnostic criteria for multiple sclerosis: 2010 revisions to the McDonald criteria. Ann Neurol.

[17] Wingerchuk DM, Banwell B, Bennett JL, Cabre P, Carroll W, Chitnis T (2015). International consensus diagnostic criteria for neuromyelitis optica spectrum disorders. Neurology.

[18] Kurtzke JF (1983). Rating neurologic impairment in multiple sclerosis: an expanded disability status scale (EDSS). Neurology.

[19] Aerts HJ, Velazquez ER, Leijenaar RT, Parmar C, Grossmann P, Carvalho S (2014). Decoding tumour phenotype by noninvasive imaging using a quantitative radiomics approach. Nat Commun.

[20] Kumar V, Gu Y, Basu S, Berglund A, Eschrich SA, Schabath MB (2012). Radiomics: the process and the challenges. Magn Reson Imaging.

[21] Wylde V, Palmer S, Learmonth ID, Dieppe P (2011). Test-retest reliability of quantitative sensory testing in knee osteoarthritis and healthy participants. Osteoarthr Cartil.

[22] Saeys Y, Inza I, Larranaga P (2007). A review of feature selection techniques in bioinformatics. Bioinformatics.

[23] Ferri FJ, Pudil P, Hatef M (1994). Comparative study of techniques for large-scale feature selection. Mach Intell Pattern Recogn.

[24] Chen C, Liaw A, Breiman L. Using random forest to learn imbalanced data. University of California, Berkeley. 2004; 110:1–12.

[25] Kim JY, Park JE, Jo Y, Shim WH, Nam SJ, Kim JH (2019). Incorporating diffusion-and perfusion-weighted MRI into a radiomics model improves diagnostic performance for pseudoprogression in glioblastoma patients. Neuro Oncol.

[26] Qu JR, Shen C, Qin JJ, Wang ZQ, Liu ZY, Guo J (2019). The MR radiomic signature can predict preoperative lymph node metastasis in patients with esophageal cancer. Eur Radiol.

[27] Lohmann P, Kocher M, Ceccon G, Bauer EK, Stoffels G, Viswanathan S (2018). Combined FET PET/MRI radiomics differentiates radiation injury from recurrent brain metastasis. NeuroImage.

[28] Orlhac F, Boughdad S, Philippe C, Hugo SB, Nioche C, Champion L (2018). A post reconstruction harmonization method for multicenter radiomic studies in PET. J Nucl Med.

[29] Sheikhan M, Bejani M, Gharavian D (2013). Modular neural-SVM scheme for speech emotion recognition using ANOVA feature selection method. Neural Comput Appl.

[30] Bennasar M, Hicks Y, Setchi R (2015). Feature selection using joint mutual information maximisation. Expert Syst Appl.

[31] Zhang B, He X, Ouyang FS, Gu DS, Dong YH, Zhang L (2017). Radiomic machine-learning classifiers for prognostic biomarkers of advanced nasopharyngeal carcinoma. Cancer Lett.

[32] Pudil P, Novovičová J, Kittler J (1994). Floating search methods in feature selection. Pattern Recognit Lett.

[33] Ghosh D, Chinnaiyan M (2005). Classification and selection of biomarkers in genomic data using LASSO. J Biomed Biotechnol.

[34] Zou H, Hastie T (2005). Regularizatin and variable selection via the elastic net. J R Statist Soc B.

[35] Guyon I, Weston J, Barnhill S, Vapnik V (2002). Gene selection for cancer classification using support vector machines. Mach Learn.

[36] Wang X, Wang D, Yao Z, Xin B, Wang B, Lan C (2018). Machine learning models for multiparametric glioma grading with quantitative result interpretations. Front Neurosci.

[37] Strumbelj E, Kononenko I (2014). Explaining prediction models and individual predictions with feature contributions. Knowl Inf Syst.

[38] Nielsen JM, Korteweg T, Barkhof F, Uitdehaag BMJ, Polman CH (2005). Overdiagnosis of multiple sclerosis and magnetic resonance imaging criteria. Ann Neurol.

[39] Matthews L, Marasco R, Jenkinson M, Küker W, Luppe S, Leite MI (2013). Distinction of seropositive NMO spectrum disorder and MS brain lesion distribution. Neurology.

[40] Lalan S, Khan M, Schlakman B, Penman A, Gatlin J, Herndon R (2012). Differentiation of neuromyelitis optica from multiple sclerosis on spinal magnetic resonance imaging. Int J MS Care.

[41] Huh SY, Min JH, Kim W, Kim SH, Kim HJ, Kim BJ (2014). The usefulness of brain MRI at onset in the differentiation of multiple sclerosis and seropositive neuromyelitis optica spectrum disorders. Mult Scler.

[42] Kim H, Lee Y, Kim YH, Lim YM, Lee JS, Woo J (2020). Deep learning-based method to differentiate neuromyelitis optica spectrum disorder from multiple sclerosis. Front Neurol.

[43] Filippi M, Bruck W, Chard D, Fazekas F, Geurts JJG, Enzinger C (2019). Association between pathological and MRI findings in multiple sclerosis. Lancet Neurol.

[44] Wang Q, Li Q, Mi R, Ye H, Zhang H, Chen B (2019). Radiomics nomogram building from multiparametric MRI to predict grade in patients with glioma: a cohort study. J Magn Reson Imaging.

[45] Liu H , Setiono R. A probabilistic approach to feature selection-a filter solution. In proceedings of the international conference on machine learning. 1996; 96:319–27.

[46] Girish C, Sahin F (2014). A survey on feature selection methods. Comput Electr Eng.

[47] Lundberg SM, Nair B, Vavilala MS, Horibe M, Eisses MJ, Adams T (2018). Explainable machine-learning predictions for the prevention of hypoxaemia during surgery. Nat Biomed Eng.

[48] Zhang B, Tian J, Dong D, Gu D, Dong Y, Zhang L (2017). Radiomics features of multiparametric MRI as novel prognostic factors in advanced nasopharyngeal carcinoma. Clin Cancer Res.

[49] Calabrese M, Poretto V, Favaretto A, Alessio S, Bernardi V, Romualdi C (2012). Cortical lesion load associates with progression of disability in multiple sclerosis. Brain.

[50] Li DK, Held U, Petkau J, Daumer M, Barkhof F, Fazekas F (2006). MRI T2 lesion burden in multiple sclerosis: a plateauing relationship with clinical disability. Neurology.

[51] Hara JH, Wu A, Villanueva-Meyer JE, Valdes G, Daggubati V, Mueller S (2018). Clinical applications of quantitative 3-dimensional MRI analysis for pediatric embryonal brain tumors. Int J Radiat Oncol Biol Phys.

[52] Karussis D (2014). The diagnosis of multiple sclerosis and the various related demyelinating syndromes: a critical review. J Autoimmun.

