# Supplementary material for: Multi-parametric MRI phenotype with trustworthy machine learning for differentiating CNS demyelinating diseases
Source: J Transl Med. 2021 Sep 6;19:377. doi: 10.1186/s12967-021-03015-w (PMC8419989; doi:10.1186/s12967-021-03015-w)
Supplement: Supplementary file 1 — Additional file 1. Appendix S1 describes radiomics features. Appendix S2 summarizes mathematical details of Multi-level Feature Selection. Appendix S3 shows results of feature selection. Table S1 describes imaging protocol for each cohort with 1.5T and 3T MRI. Table S2 shows the parameter settings for feature extraction. [file 12967_2021_3015_MOESM1_ESM.docx]

Additional file 1:

# Appendix S1 Description of radiomics features

Intensity features were calculated based on the first-order statistics of the image intensity distribution. Texture features were computed with higher-order texture matrices such as Gray Level Co-occurrence Matrix (GLCM) and Gray Level Size Zone Matrix (GLSZM) to quantify lesion heterogeneity. Filter-based features, including Laplace of Gaussian (LoG) and wavelet features were extracted from filtered images to enhance specific parts of images, such as sharp edges or fine texture.

# Appendix S2 Mathematical details of Multi-level Feature Selection

Multi-Level Feature Selection composed of univariate selection of robust features, relevant features and multivariate selection of discriminative features, as shown in **Algorithm 1** below. The feature selection was performed on the training dataset $X \in\mathbb{R}^{p*q}$, and required a parameter d as max number of features to select. Specifically, 1) in univariate selection of robust features, we used Wilcoxon rank-sum test to select features that were robust across 1.5T MRI and 3T MRI in both MS cohort ($X_{ms1.5}$ and $X_{ms3}$) and NMO cohort ($X_{nmo1.5}$and $X_{nmo3}$). 2) In univariate selection of relevant features, we performed Wilcoxon rank-sum test to choose features with significant statistical differences between MS cohort ($X_{ms}$) and NMO cohort ($X_{nmo}$). The cut-off p-value was set to 0.05. 3) With selected features $F^{u}$ from univariate analysis, we further applied sequential forward selection in multivariate analysis to obtain the final feature set $F^{m}.$


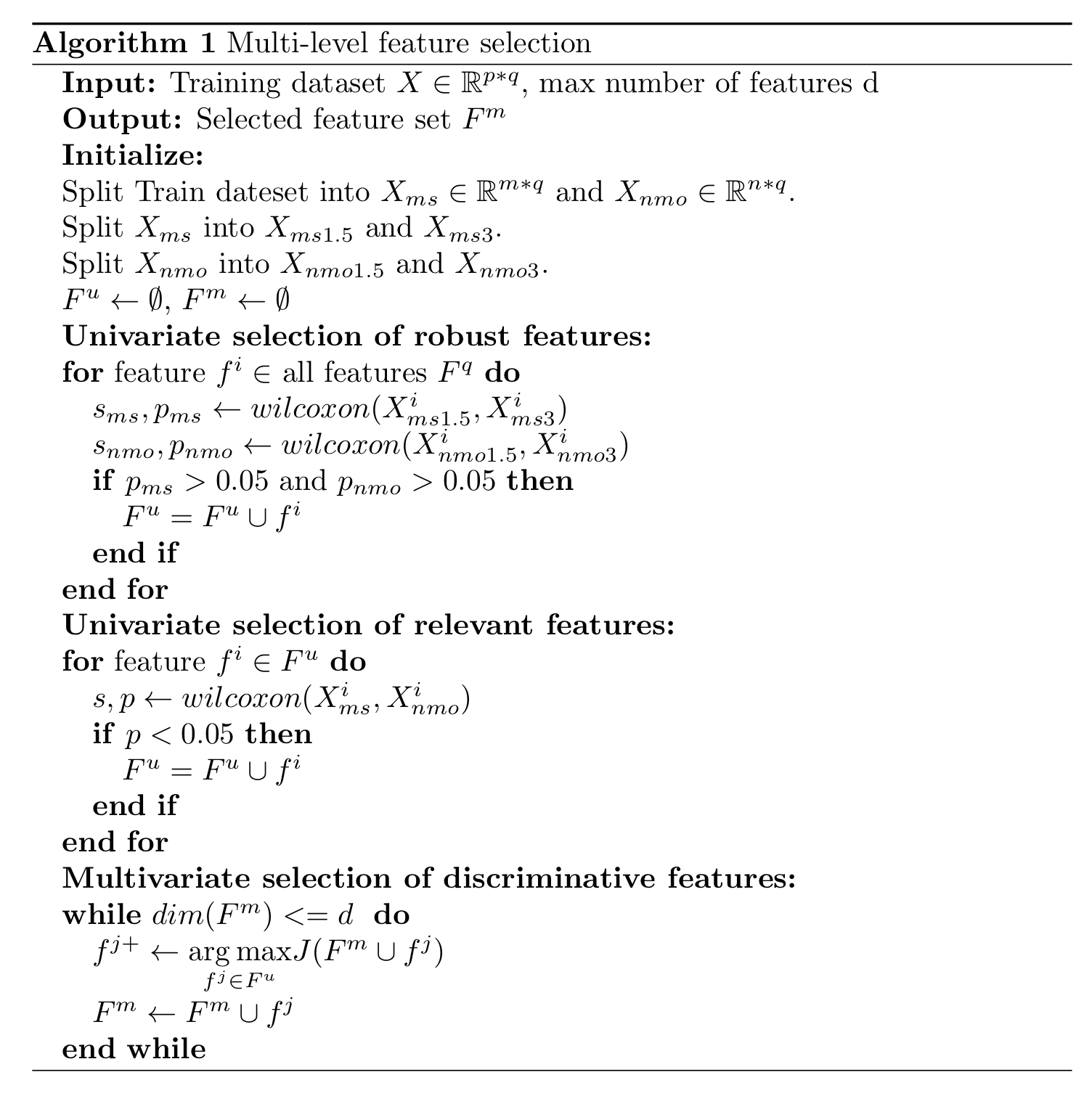


# Appendix S3 Results of feature selection

In univariate feature selection, 450 T2 and 117 T1-MPRAGE robust features across 1.5T and 3T MR images were firstly selected from 2236 radiomic features. After that, 313 T2 and 86 T1-MPRAGE discriminative features were identified from robust features for differentiation of MS and NMO. Seven T2 features, four T1-MPRAGE features and one clinical feature were selected from 313 T2, 86 T1-MPRAGE features and four clinical features respectively, to form the corresponding preliminary phenotypes. From 12 fused features from T2, T1-MPRAGE and clinical phenotypes, the multi-parametric phenotype was established with three T2, four T1-MPRAGE and one clinical feature.

# Table S1. Imaging protocol for each cohort with 1.5T and 3T MRI

| Parameter | 1.5T MRI cohort | 3T MRI cohort |
| --- | --- | --- |
| Field strength | 1.5T | 3T |
| MRI model | Sonata; Siemens Medical Systems, Erlangen, Germany | Siemens Magnetom Trio Tim System, Munich, Germany |
| Head receiver coil | 8-channel | 12-channel |
| **Sequence: T2** |  |  |
| TR / TE | 5500 / 94ms | 5000 / 87 ms |
| number of signals acquired | 3 | 1 |
| echo train length | 11 | 15 |
| FOV | 240 mm × 210 mm | 256 mm × 256 mm |
| matrix size | 256 × 224 | 256 × 256 |
| number of slices | 30 | 30 |
| section thickness | 4 mm | 4 mm |
| intersection gap | 0.4 mm | 0.4 mm |
| **Sequence: T1-MPRAGE** |  |  |
| TR / TE | 1970 / 3.90 ms | 1600 / 2.13 ms |
| TI | 1100 ms | 1000 ms |
| flip angle | 15° | 9° |
| FOV | 250 mm × 219 mm | 256 mm × 224 mm |
| matrix size | 256 × 256 | 256 × 224 |
| slice thickness | 1.7 mm | 1.0 mm |
| voxel dimensions | 0.5 mm × 0.5 mm × 1.7 mm | 1.0 mm × 1.0 mm × 1.0 mm |

Abbreviations: TE = echo time; TR = repetition time

# Table S2. The parameter settings for feature extraction

|  | **Types** | **Parameters** |
| --- | --- | --- |
| Filtering | No filter | N/A |
|  | LoG filter | Sigma = 2mm, 3mm, 4mm and 5mm |
|  | Wavelet filter | Decompositions = LLL, LLH, LHL, LHH, HLL, HLH, HHL, HHH;  Wavelet_type = ‘coif1’ |
| Feature extraction | Intensity | Binwidth=5 |
|  | GLCM | distance=1;  symmetrical=True;  weightedNorm=None |
|  | GLRLM | weightedNorm=None |
|  | GLSZM | N/A |
|  | NGTDM | N/A |
